# IGF-1 secreted by mesenchymal stem cells affects the function of lymphatic endothelial progenitor cells: a potential strategy for the treatment of lymphedema

**DOI:** 10.3389/fgene.2025.1584095

**Published:** 2025-05-21

**Authors:** Zekuan Xue, Dongdong Yang, Zhiwei Jin, Yijie Li, Yunfei Yu, Xinchun Zhao, Yongzhou Huang, Shengqiu Jia, Tong Zhang, Guilin Huang, Jixue Hou

**Affiliations:** ^1^ School of Medicine, Shihezi University, Shihezi, China; ^2^ Department of Thyroid and Breast Surgery, The First Affiliated Hospital of Shihezi University, Shihezi, China; ^3^ Department of Urology, Weihui People’s Hospital, Xinxiang, China

**Keywords:** mesenchymal stem cells, endothelial progenitor cells, proliferation, insulin-like growth factor 1, lymphedema

## Abstract

Mesenchymal stem cells (MSCs) can participate in lymphangiogenesis through paracrine effects, while lymphatic endothelial progenitor cells (LEPCs), a subpopulation of endothelial progenitor cells (EPCs), can differentiate into mature lymphatic endothelial cells, thereby influencing lymphatic function. In the present study, we investigated the mechanism by which MSCs regulate the activity of LEPCs through paracrine effects and preliminarily explored the possibility of the two types of cells working together to treat lymphovascular diseases. After isolation of MSCs and LEPCs from the bone marrow of C57BL/6 J mice, *in vitro* experiments verified that insulin-like growth factor 1 (IGF-1) secreted by MSCs activated the PI3K/Akt/mTOR pathway to promote the proliferation of LEPCs; IGF-1 decreased the rate of apoptosis and affected the cycle progression of LEPCs and the nucleotide metabolism levels. The therapeutic efficacy of combined transplantation of MSCs and LEPCs was shown to be superior to that of transplantation of LEPCs alone in murine hindlimb lymphedema models. These results suggest that MSCs significantly promote the proliferation of LEPCs through the activation of the PI3K/Akt/mTOR pathway in LEPCs by secreting IGF-1, and that IGF-1 also inhibits apoptosis and regulates cell metabolism. Combined transplantation of MSCs and LEPCs provides an experimental rationale and potential strategy for cell therapy in lymphedema.

## 1 Introduction

Cancer patients who have undergone lymph node dissection or radiotherapy often develop lymphedema due to varying degrees of lymphatic destruction, which can seriously affect their recovery and quality of life (Brix et al., 2021). Lymphedema can be treated surgically or non-surgically. Surgical treatments offer some benefit to patients with oedema but are more invasive, while non-surgical treatments often need to be repeated due to poor results, which can lead to worsening of the oedema. In recent years, stem cell transplantation therapy has shown promising application prospects in many disease areas, and the targeted induction of stem cells during lymphangiogenesis to promote the formation of lymphatic return channels may be a new strategy for the treatment of lymphedema (Ismail et al., 2018; Alvites et al., 2022).

Mesenchymal stem cells (MSCs) are stem cells that are self-renewing, highly proliferative and capable of differentiating into a wide range of tissue cells (Pittenger et al., 1999) and can be isolated from a variety of tissues. In recent years, relevant studies have shown that MSCs influence the function of other cells or tissue regeneration and repair through their secretory function, demonstrating their potential application value. MSC-secreted exosomes have been shown to promote endothelial cell angiogenesis *in vitro* and in immunodeficient mice (Liang et al., 2016); MSC-derived exosomes reverse hydrogen peroxide-induced endothelial cell apoptosis and enhance granulation tissue formation in wounds of diabetic mice (Zhao et al., 2023). In addition, MSC-derived extracellular vesicles under hypoxic conditions enhanced lymphangiogenesis by promoting proliferation, migration and activation of Akt signalling in lymphatic endothelial cells (Yang et al., 2022). It has also been shown that injection of MSCs at the site of vascular injury can secrete growth factors to accelerate angiogenesis, which promotes the angiogenic capacity of endothelial cells by secreting vascular endothelial growth factor (VEGF) (An et al., 2018).

Endothelial progenitor cells (EPCs) are a class of precursor cells that can differentiate into mature endothelial cells, and they have been extensively studied in the field of angiogenesis since their discovery by Asahara and colleagues (Asahara et al., 1997; Wang et al., 2016; Jiang et al., 2021). There are currently two major subpopulations of EPCs, vascular endothelial progenitor cells (VEPCs) and lymphatic endothelial progenitor cells (LEPCs) (Friedrich et al., 2006; DiMaio et al., 2016). Although the 2 cells share some surface markers (e.g., CD34), LEPCs specifically express high levels of key factors for lymphatic endothelial differentiation such as VEGFR-3 and LYVE-1, and LEPCs can differentiate into lymphatic endothelial cells and regulate lymphangiogenesis (Tan et al., 2014; Campbell et al., 2021).

MSC conditioned medium (MSC-CM) has been shown to stimulate lymphangiogenesis (Robering et al., 2018). In addition, MSCs can secrete a variety of lymphatic growth factors, including insulin-like growth factor-1 (IGF-1), epidermal growth factor (EGF), VEGF, and hematopoietic growth factor (HGF) (Chen et al., 2008; Robering et al., 2018), which may be involved in lymphangiogenesis (Takeda et al., 2015; Lee et al., 2016). Among these, IGF-1 can induce phosphorylation of the protein kinase Akt, which promotes proliferation and migration of human or mouse lymphatic endothelial cells and is directly or indirectly involved in lymphatic vessel formation (Björndahl et al., 2005). Our previous studies have shown that MSCs can adhere to EPCs and stimulate the proliferation of EPCs by secreting IGF-1 (Hou et al., 2017; Guo et al., 2018). It has also been shown that IGF-1 can stimulate the clonogenic and angiogenic capacity of EPCs (Humpert et al., 2008; Kamprom et al., 2016). Therefore, we speculated whether IGF-1 has a similar effect on LEPCs, which are a subpopulation of EPCs.

MSCs offer additional possibilities in the field of stem cell therapy and have received much attention for their interaction with other types of stem cells through their own secretory function. LEPCs can differentiate into lymphatic endothelial cells, which are thought to contribute to developmental and postnatal lymphangiogenesis (Park et al., 2011; Kazenwadel and Harvey, 2018). There are fewer reports on the mechanism of interaction between MSCs and LEPCs, so to explore the mechanism and the possibility of stem cell co-transplantation for the treatment of lymphedema, in this paper, we investigated the alteration of the biological behaviour of LEPCs mediated by cytokines secreted by MSCs by isolating MSCs and LEPCs from the bone marrow of mice, and we evaluated the effect of co-transplantation of the two types of cells in the treatment of lymphedema to further deepen the understanding of their interaction.

## 2 Materials and methods

### 2.1 Animal preparation

The healthy C57BL/6J mice utilised in this study were procured from Sipeifu Biotech (Beijing, China) and subsequently housed in the Animal Experimentation Centre of Shihezi University (Shihezi, China) under controlled laboratory conditions (temperature of 22°C; 12 h light/12 h dark cycle, 55% relative humidity, and up to four animals per cage). The mice were provided with unrestricted access to food and water. Experiments began 1 week after animals were acclimated to the environment. A total of 20 female C57BL/6J mice (wild-type; body weight 13–15 g; age 4 weeks) were used for primary cell collection. In addition, 30 female C57BL/6J mice (wild-type; body weight 16–18 g; age 6 weeks) were used to establish the model of lymphedema. Every effort was made to minimise suffering and sacrifice fewer animals. The experiments and manipulation of the mice in this study were conducted in accordance with the requirements of the Bioethics Committee of Shihezi University (A2024-408) on the handling of laboratory animals and animal welfare.

### 2.2 Isolation and culture of MSCs

Following the anaesthetisation of the mice by intraperitoneal injection of 1% sodium pentobarbital, the skin of the hind limbs was removed bilaterally, and the mice were euthanised using the cervical dislocation method. Thereafter, the mice were immersed in 70% alcohol for 1 min. The femurs and tibias were removed using surgical instruments on a sterile operating table, and the muscles and connective tissues were completely removed from the bones (Supplementary Figure S1). Bones were rinsed with phosphate buffered saline (PBS) (Solarbio, China) and the epiphyses removed to expose the marrow cavity. A 1 mL syringe was used to aspirate the configured MEM-α medium (Shanghai BasalMedia Technologies, China), which consisted of 10% fetal bovine serum (FBS) (Omnimabs, United States), 100 U/mL penicillin and 100 μg/mL streptomycin (Solarbio, China). Bone marrow cells were gently flushed out and collected using a syringe. Cells remaining after removal of erythrocytes using erythrocyte lysate (Tiangen, China) were cultured in 10 cm dishes with MEM-α medium in a humidified incubator (Thermo, United States) at a temperature of 37°C with 95% air and 5% CO_2_. The medium was replaced with fresh MEM-α after 24 h and every 2 days thereafter. When the fusion of MSCs reached 80%–90%, the cells were dissociated using 0.25% trypsin-EDTA (NCM Biotech, China) and passaged in a 1:2 ratio.

### 2.3 Phenotyping of MSCs by flow cytometry

To detect surface markers of MSCs, 1 × 10^6^ cells were incubated with fluorescein isothiocyanate (FITC)-conjugated anti-mouse CD44 antibody (Invitrogen, United States), APC-conjugated anti-mouse CD29 antibody (Biolegend, United States) and PerCP-cyanine5.5-conjugated anti-mouse Sca-1 antibody (Invitrogen, United States). 1 × 10^6^ cells were incubated with APC-conjugated anti-mouse CD45 antibody (Invitrogen, United States) and PE-conjugated anti-mouse CD11b antibody (Invitrogen, United States). Cells were incubated with antibodies for 30 min at 4°C in the dark, then washed with PBS to remove unbound antibodies and analysed within 1 h using flow cytometer (Agilent, United States).

### 2.4 Multi-directional differentiation of MSCs

MSCs were inoculated into 6-well plates coated with 0.1% gelatin (Solarbio, China) at a density of 150,000 cells per well. When the fusion of the cells reached 90%–100%, the cells were cultured for 2–3 weeks using mouse bone marrow mesenchymal stem cell lipogenic induced differentiation medium (Procell, China) or osteogenic induced differentiation complete medium (Procell, China) according to the protocols. Lipogenic and osteogenic differentiation of the cells was analysed by staining with Oil Red O (Procell, China) and Alizarin Red (Procell, China).

For chondrogenic induction, MSCs were subjected to chondrogenic differentiation in standard precipitation cultures to detect proteoglycans. Centrifuge a total of 400,000 cells at 250 x g for 4 min at room temperature to form a precipitate. Fresh chondrogenic induction and differentiation medium (Procell, China) was changed every 2 days to promote the growth of cell depositions. After 3 weeks of incubation, the precipitates were cut into sections and stained with Alcian Blue staining solution (Servicebio, China) to detect the presence of proteoglycans.

### 2.5 Isolation and culture of LEPCs

Referring to Wang et al. (Wang et al., 2017), the CD34^+^VEGFR-3^+^ EPCs population with lymphatic potential were used as LEPCs.The femurs and tibias of the mice were removed using the same surgical procedure as for MSCs harvesting, and the bones were then rinsed with PBS and the bone marrow cavity exposed. Bone marrow cells were rinsed and collected with Endothelial Basal Medium-2 (EBM-2) (Lonza, Switzerland) and the mononuclear cells were isolated from them by density gradient centrifugation with Percoll solution (GE Healthcare, United States). CD34^+^ cells in mononuclear cells were isolated by positive selection using anti-CD34 microbeads (Miltenyi Biotec, Germany) and a magnetic cell sorter (Miltenyi Biotec, Germany) according to the manufacturer’s recommended protocol. Isolated CD34^+^ cells were cultured in 60 mm dishes using EBM-2 containing 10% FBS, 100 U/mL penicillin, 100 μg/mL streptomycin and 10 ng/mL recombinant VEGF-C (Abcam,UK). VEGFR-3^+^ cells were isolated from CD34^+^ cells by positive selection using anti-VEGFR-3 microbeads (Miltenyi Biotec, Germany) after 3 days of cell culture according to the manufacturer’s recommended procedure. Sorted CD34^+^ VEGFR-3^+^ cells were inoculated with configured EBM-2 medium and continued to be cultured in gelatin-coated 60 mm dishes, with fresh medium changed every 48 h.

### 2.6 Characterisation of LEPCs

The isolated LEPCs were cultured for approximately 14 days, after which the cells were identified by a combination of specific surface antigen expression and functional properties. To understand the expression of cell surface markers, immunostaining was performed using rat anti-CD34 antibody (1:100, Invitrogen, United States) and rabbit anti-VEGFR-3 antibody (1:200, Invitrogen, United States). Cells were incubated with goat anti-rat Cy3 (Cyanine3) (1:1000, Invitrogen, United States) or goat anti-rabbit FITC (1:500, Invitrogen, United States) conjugated secondary antibodies and finally stained with 4′, 6-diamidino-2-phenylindole (DAPI) (Servicebio, China) for nuclear staining, and positive staining was detected by fluorescence microscopy (Olympus, Japan).

To determine the endothelial phenotype of the cells, DiI-Acetylated Low Density Lipoprotein (DiI-acLDL) (1:200, MKbio, China)uptake experiments were performed. LEPCs were stained with DiI-labelled acLDL for 4 h in the dark at 37°C. Cells were fixed with 4% formaldehyde for 10 min and then stained with FITC-conjugated ulex europaeus agglutinin 1 (UEA-1) (1:200, MKbio, China) for 1 h in the dark. The cells were photographed under a fluorescence microscope.

### 2.7 Tube formation assay

To verify the ability of LEPCs to form lymphatic capillary-like structures, matrix gel (Corning, United States) was stored at 4°C overnight to thaw, and then spread uniformly into ice-cold 24-well plates at a dose of 50 µL/well, and the well plates were placed at 37°C for 1 h to allow the matrix gel to solidify. LEPCs were inoculated onto the matrix gel at a density of 200,000 LEPCs per well and incubated at 37°C for approximately 3 h. Also to verify the activity of the cells forming the pipeline, each well was incubated for 30 min at 37°C in a dark environment after adding 100 µL of Calcein AM assay working solution (Servicebio, China). Replace with fresh cell culture medium to ensure adequate hydrolysis of intracellular calcein AM. Microscopic images of tube formation were taken using fluorescence microscopy.

### 2.8 Co-culture of MSCs and LEPCs

MSCs and LEPCs were inoculated into the upper and lower chambers, respectively, of a transwell system (Corning, United States) with polycarbonate membrane inserts at a 1:1 ratio of cell number, and different well specifications were used according to different experimental requirements. In the experimental group, LEPCs were inoculated into the lower chamber, while MSCs were inoculated into the upper chamber. In the control group, only LEPCs were inoculated into the lower chamber and the upper chamber contained only basal medium for MSCs.

### 2.9 CCK-8 assay

The proliferative activity of LEPCs under different culture conditions (LEPCs co-cultured with MSCs, LEPCs after stimulation with different concentrations of IGF-1 (Abcam, UK), LEPCs with antibody neutralising agents or pathway inhibitors) was determined using CCK-8. Briefly, after co-culturing LEPCs with MSCs at a density of 6000 cells per well in a 96-well transwell system for varying periods of time, the upper chambers containing the MSCs were removed and 10 µL of CCK-8 reagent (APExBIO, United States) and 90 µL of EBM-2 were added to each lower chamber. CCK-8 reagents and LEPCs were incubated for 2 h in a dark environment and then the absorbance was measured at 450 nm using an enzyme-linked immunoassay detector (Thermo Fisher Scientific, United States).

### 2.10 EdU staining

MSCs were co-cultured with LEPCs at a density of 140,000 cells per well in a 12-well transwell system for 72 h after which the upper chamber containing MSCs was removed. LEPCs in the lower chamber were fixed and stained using the EdU Imaging Kit (cy3) (APExBIO, United States) configured with working reagents according to the recommended procedure. Cell nuclei were stained with Hoechst 33,342 solution (5 μg/mL) from the kit. Cells were observed and photographed under a fluorescence microscope.

### 2.11 ELISA

MSCs and LEPCs were cultured at a density of 100,000 per dish in 60 mm dishes with medium without FBS and growth factors for 24 h. The medium was collected by centrifugation (4°C, 6000 rpm, 5 min). IGF-1 was detected in the culture medium using an IGF-1 ELISA kit (Multisciences, China) according to the manufacturer’s protocol.

### 2.12 Detection of apoptosis and cell cycle by flow cytometry

The number of apoptotic cells was detected using the Annexin V-FITC/PI Apoptosis Kit (MULTISCIENCES, China). After rinsing the cells with PBS, the cells are suspended by adding binding buffer according to the manufacturer’s protocol. Cells were then labelled with Annexin V-FITC and PI for 10 min at room temperature in the dark according to the protocol. Afterwards, cells were detected with FACS Canto II CytoFLEX (BD, United States) and analysed with FlowJo 10.8.1 (BD, United States). For cell cycle analysis, cells were fixed using pre-cooled 75% ethanol overnight then washed twice with PBS, 100 µL PBS was used to re-suspend the cells and 2 µL of RNaseA (10 mg/mL) (Servicebio, China) was added and incubated for 30 min at 37°C. Cells were stained using propidium iodide (100 μg/mL) (Servicebio, China) for 10 min in the dark. Cycle distributions were detected with a BD FACS Canto II CytoFLEX (BD, United States) and determined using ModFit software (Verity Software House, United States).

### 2.13 Western blotting assay

The total proteins of LEPCs were extracted using RIPA buffer (Solarbio, China) and phenylmethanesulfonyl fluoride (PMSF) (Solarbio, China) and quantified using BCA analysis kit (Solarbio, China). 10% SDS-polyacrylamide gels (Servicebio, China) were used for loading and electrophoresis. The isolated proteins were then transferred to a polyvinylidene difluoride membrane (PVDF) (Millipore, United States). PDVF membranes were incubated at 4°C with rabbit anti-p-Akt (Ser473) (1:1000, CST, United States), rabbit anti-p-S6 (1:1000, CST, United States), rabbit anti-Akt (1:1000, CST, United States), rabbit anti-S6 (1:1000, CST, United States), rabbit anti-Bax (1:2000, Proteintech, China), rabbit anti-Bcl-2 (1:2000, Proteintech, China), rabbit anti-caspase3 (1:2000, Proteintech, China), rabbit anti-RRM1 (1:1000, CST, United States), rabbit anti-RRM2 (1:1000, CST, United States) and mouse anti-GAPDH primary antibody (1:2000, zsgb bio, China) were incubated overnight. Subsequently, the membrane was subjected to an incubation with goat anti-rabbit (1:2000, Zsgb Bio, China) or goat anti-mouse (1:2000, Zsgb Bio, China) for 60 min at room temperature. Immunoreactive bands were observed using an ECL kit (Biosharp, China) and analysed using ImageJ 1.5 software (NIH, Bethesda, MD, United States).

### 2.14 RNA-seq

Total RNA was extracted from the cells using TRIzol reagent (Invitrogen, United States) and the concentration and purity of total RNA was analysed using Bioanalyzer 2100 (Agilent, United States). After purification, the mRNA was fragmented using ionic disruption at elevated temperatures. The cut RNA fragments were then reverse transcribed into cDNA using SuperScript II reverse transcriptase (Invitrogen, United States), and the first-strand cDNA was used as a template for second-strand cDNA synthesis. Upon completion of library construction, PCR amplification was used for library fragment enrichment, followed by library selection based on fragment size and library quality control using the Agilent 2100 Bioanalyzer. Finally, the libraries were subjected to paired-end (PE) sequencing using Next-Generation Sequencing (NGS) based on the Illumina sequencing platform at Shanghai Bioprofile Technology Company Ltd.

### 2.15 LC-MS targeted metabolomics analysis

Following a 72 h exposure to IGF-1, the LEPCs were subjected to a series of procedures. Initially, the cells were washed with pre-cooled PBS. Thereafter, they were collected on ice using a cell scraper bar (Corning, United States). Finally, the cells were subjected to centrifugation in order to remove the upper layer of culture medium. Samples were vortex mixed with 1000 μL of pre-cooled 80% methanol solution, sonicated on ice for 20 min, allowed to stand at −20°C for 1 h, centrifuged at 16,000 g for 20 min at 4°C and the supernatant was collected and lyophilised. Samples were redissolved in 80 μL of pre-cooled 50% aqueous methanol and the supernatant was centrifuged at 20,000 g for 15 min at 4°C in preparation for mass spectrometric injection analysis. LC-MS/MS analyses were performed by Shanghai Bioprofile Technology Company Ltd. and were separated by Shimadzu Nexera X2 LC-30AD high performance liquid chromatography (HPLC) and analysed by mass spectrometry (MS) using a QTRAP 6500 mass spectrometer (AB SCIEX, United States).

### 2.16 Establishment of a mouse hindlimb lymphedema model

The model was built with reference to other scholars’ methods (Wiinholt et al., 2019)and partially modified. The mice were anaesthetised by intraperitoneal injection of sodium pentobarbital, and the surgery was started after successful anaesthesia. Lymph nodes and lymphatic vessels in the left hind limb were labelled by subcutaneous injection of patent blue V (Acid blue 1, MCE, United States) into the toes of the mice. As much hair as possible was removed from the hind limbs. Surgery is performed in a dedicated surgical suite to ensure sterile surfaces. A circular skin incision was made in the femoral region and the marked popliteal lymph node (PLN), two lymphatic vessels distal to the lymph node (DLV1 and DLV2) and one lymphatic vessel proximal to the lymph node (PLV) were dissected (Supplementary Figure S2). Popliteal lymph nodes were excised and proximal and distal lymphatic vessels were ligated with nylon sutures (Johnson & Johnson, United States). Remove as much fatty tissue as possible from the groin area and rinse the surgical site with sterile saline. After confirming that there is no bleeding, the skin edges at both ends of the incision are secured to the myofascia with nylon sutures. The mice were housed in a warm, clean rearing environment and the wounds were observed for signs of infection. The thickness of the left footpad was measured before surgery and on day 1 after surgery using a scalar disc gauge, while the thickness of the right footpad was measured as a control to assess the degree of oedema and then every 3 days thereafter. To avoid measurement errors, the mouse holder and the surveyor maintain a fixed posture and position.

### 2.17 Animal groups and cells transplantation

To observe the alleviating effect of MSCs and LEPCs on lymphedema, mice were grouped in experiments. The No operation group consisted of five unoperated mice; the PBS control group, the LEPC transplantation group and the MSC combined LEPC transplantation group each consisted of five operated mice. PBS or cells were injected into the surgical site with a 27-gauge needle on day 7 after surgery (0.1 mL of PBS in the control group, 1,000,000 LEPCs in the LEPC transplantation group, and 500,000 MSCs mixed with 500,000 LEPCs in the co-transplantation group), and the cells were all mixed with 0.1 mL of PBS. Assessment of oedema relief included measurement of left footpad thickness and immunohistochemical analysis of lymphatic vessels.

### 2.18 Immunohistochemical detection of lymphatic vessels

At 19 days after cell transplantation, tissue samples from the left hind limb were removed from each group of mice after euthanasia and histological assessment was performed using sections of the largest portion of the femoral region (n = 5). Samples were fixed in 4% paraformaldehyde solution (Servicebio, China) for 24 h, then embedded in paraffin and cut into 4 µm sections. Sections were dewaxed and antigenically repaired in citric acid antigen repair solution (Servicebio, China) and blocked with goat serum. Sections were incubated with rabbit anti-LYVE-1 (1:2000, Servicebio, China) used to identify lymphatic vessels overnight at 4°C and with HRP-labelled goat anti-rabbit (1:500, Servicebio, China) for 50 min at room temperature. The development of colour was achieved through the utilisation of DAB (Servicebio, China), followed by staining with hematoxylin (Servicebio, China). The samples were then dehydrated and blocked, after which observation and photography were conducted using a microscope (Nikon, Japan). The quantification of the number and area of lymphatic vessels was conducted using ImageJ 1.5 software (NIH, Bethesda, MD, United States).

### 2.19 Statistical analysis

The normality of the sample distribution was confirmed by the Shapiro-Wilk test. Statistical comparisons between the two groups were made using the Student’s t-test; ANOVA was performed on multiple groups, followed by the LSD-t-test. Data are expressed as mean ± standard error of the mean (SEM). p < 0.05 was considered statistically significant. Data were analysed using IBM SPSS Statistics 26 (IBM, United States). Plots were generated using GraphPadprism 9.0 software (GraphPad Software, United States).

## 3 Results

### 3.1 Characterization of MSCs

**FIGURE 1:**
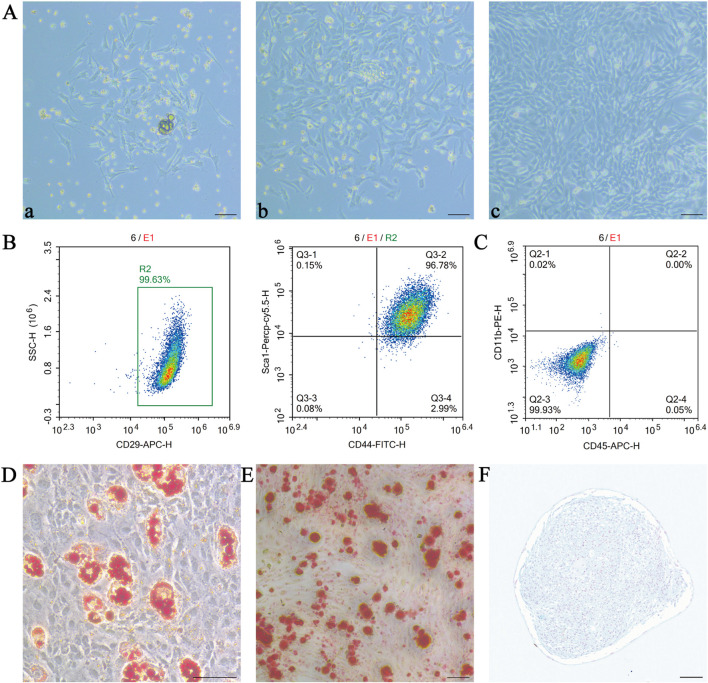
Characterisation of mesenchymal stem cells (MSCs). We isolated and cultured MSCs from mouse bone marrow without any treatment and studied their relevant characteristics. **(A)** Representative photographs of MSCs extracted at different time points during culture at 24 h **(a)**, 48 h **(b)** and after passaging **(c)**; **(B,C)** Flow cytometric detection of positive markers CD29, CD44 and Sca-1 and negative markers CD45 and CD11b; Oil red O staining **(D)**, alizarin red staining **(E)** and alcian blue staining **(F)** were used to verify the lipogenic, osteogenic and chondrogenic differentiation ability of MSCs, respectively. scale bar 50 µm in **(A,D,E)**; scale bar 100 µm in **(F)**. MSCs, mesenchymal stem or stromal cells.

MSCs derived from mouse bone marrow showed multiple adherent colonies after 24 h of culture (Figure 1Aa), and the colonies continued to grow after 48 h of culture (Figure 1Ab), and the morphology of the cells was predominantly long spindle-shaped with radioactive growth. After 10–14 days of culture, the fusion of MSCs reached 80%–90%, and the passaged cells were in good adherence and proliferation status, with uniform distribution and swirling growth (Figure 1Ac).

Flow cytometry results showed that the cultured MSCs expressed high levels of CD29, CD44 and Sca-1 (Figure 1B), but were negative for the haematopoietic markers CD45 and CD11b (Figure 1C). MSCs were tested for their ability to differentiate after induction of multidirectional differentiation, and red lipid droplets could be seen in the cells after staining with oil red O (Figure 1D); the cells were stained with alizarin red S, and calcium deposits were present on the surface of the red areas shown, indicating that the cells were undergoing osteogenic differentiation (Figure 1E). The cartilage spheres formed by MSCs were sectioned by paraffin embedding and then stained using Alcian Blue, the stain reacted with acidic mucopolysaccharides within the cartilage tissue in blue colour, which indicated that MSCs had differentiated into chondrocytes (Figure 1F). These data indicate that the cultured cells have typical characteristics of MSCs.

### 3.2 Characterisation of lymphatic endothelial progenitor cells

**FIGURE 2:**
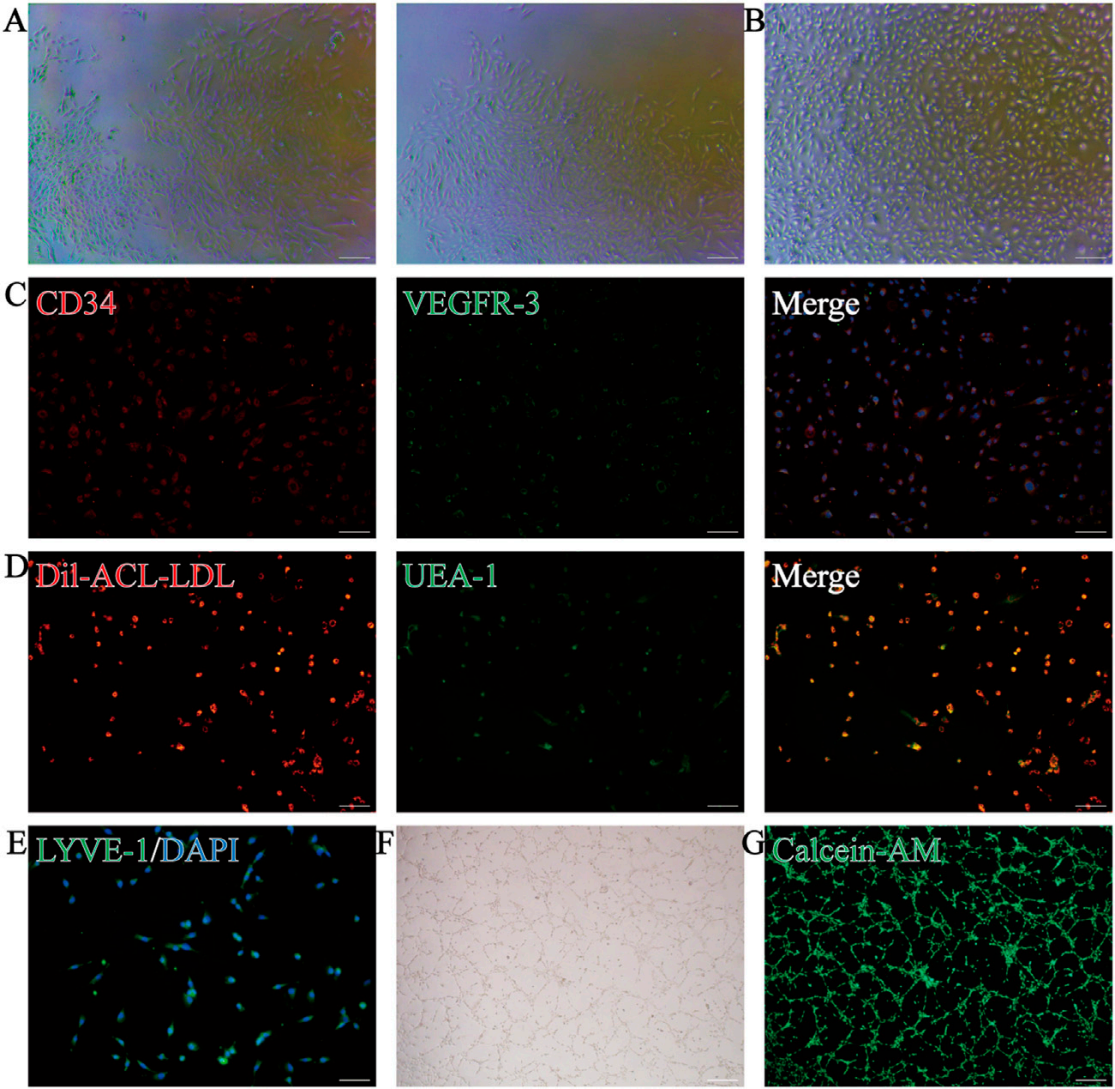
Characterisation and differentiation capacity of lymphatic endothelial progenitor cells (LEPCs). **(A)** LEPCs isolated from mouse bone marrow mononuclear cells with colonies formed after 7 days in culture; **(B)** Microscopic image of LEPCs after 2 weeks of incubation; **(C)** Immunostaining to detect the expression of CD34 (red) and VEGFR-3 (green) in the cells, and DAPI (blue) to re-stain the nuclei; **(D)** Uptake of Dil-Ac-LDL (red) and binding of UEA-1 (green); these positive cells represent the endothelial progenitor cell phenotype; **(E)** Immunofluorescence detection of LYVE-1 (green) expression in cells and DAPI (blue) staining of nuclei; **(F)** LEPCs formed tube-like structures in the matrix gel and were stained with calcein AM (green) **(G)** to identify cell viability. scale bar 50 µm in A-E,scale bar 100 µm in F,G; VEGFR-3, vascular endothelial growth factor receptor-3; LEPCs, lymphatic endothelial progenitor cells; LYVE-1, lymphatic vessel endothelial hyaluronan receptor-1.

Isolated CD34^+^VEGFR-3^+^ cells were cultured on day 7 and the cell colonies showed long, shuttle-shaped, fish-like growth (Figure 2A). Cell fusion reached 80%–90% by day 14 in culture, and the cells were well adherent to the wall after passaging, with cobblestone-like uniform growth, similar to endothelial cells (Figure 2B).

To verify the purity of the sorted cells and their ability to differentiate into lymphatic endothelial cells, we used immunofluorescence to detect the stem cell marker CD34 and the lymphatic endothelial cell marker VEGFR-3, which showed that the cells expressed both CD34 and VEGFR-3 (Figure 2C). Consistent with the phenotype of EPCs, the cells were also positive for both UEA-I-FITC binding and Dil-Ac-LDL uptake (Figure 2D). After 2 weeks in culture, the cells expressed the lymphatic endothelial cell marker LYVE-1 (Figure 2E), suggesting that the cultured cells have the potential to differentiate into lymphatic endothelial cells. More importantly, cells cultured on matrix gel were able to form stable tubular structures (Figure 2F), and calcium xanthophyll staining suggested better cell viability (Figure 2G). Taken together, these results suggest that the cells we isolated and cultured were LEPCs with the potential to differentiate into lymphatic endothelial cells.

### 3.3 IGF-1 secretion by MSCs promotes proliferation and inhibits apoptosis of LEPCs

**FIGURE 3:**
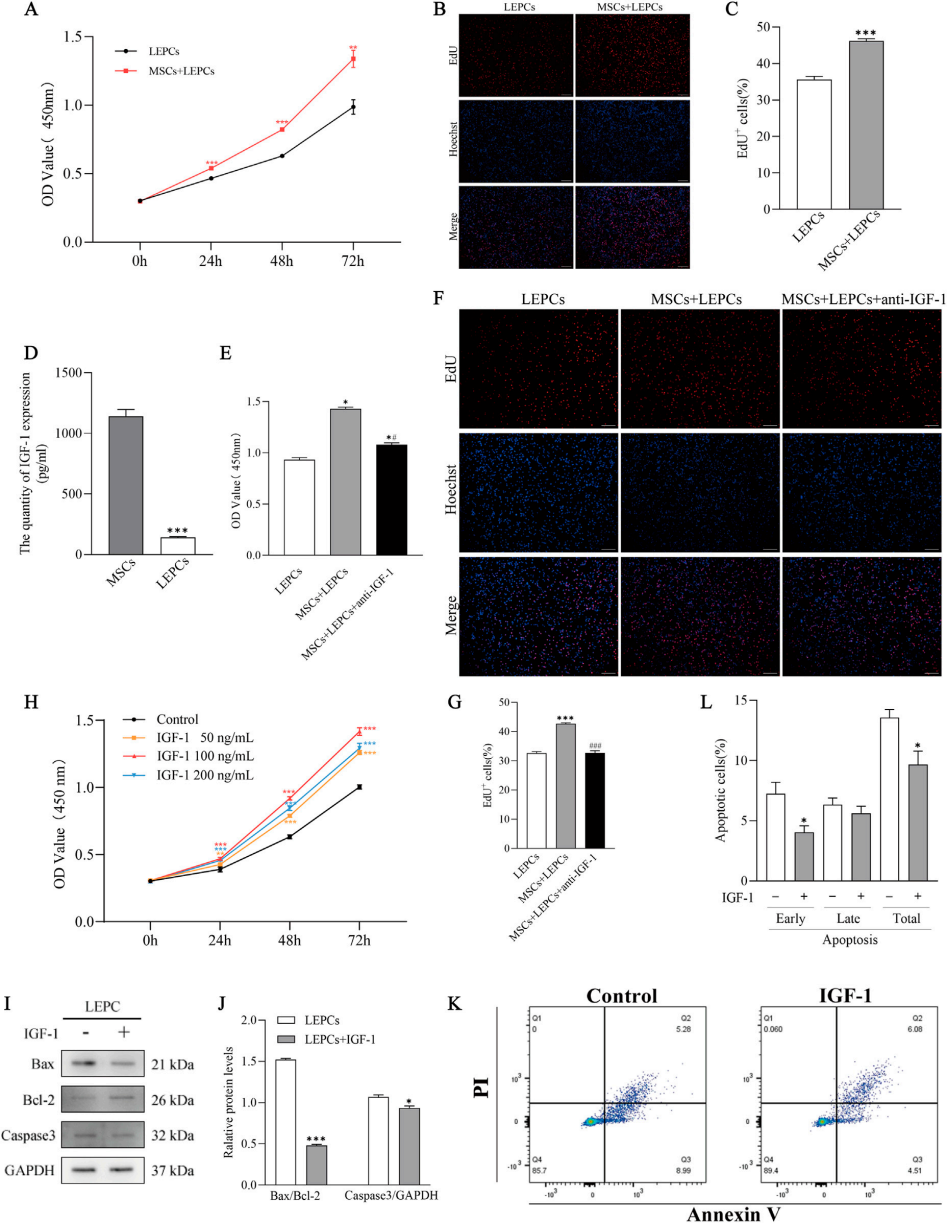
Insulin-like growth factor 1 (IGF-1) secreted by MSC promotes LEPC proliferation and inhibits apoptosis. **(A)** LEPCs were cultured alone or co-cultured with MSCs for 0 h, 24 h, 48 h or 72 h. Absorbance of LEPCs was measured by CCK-8. **p < 0.01,***p < 0.001 *versus* LEPCs group; **(B,C)** LEPCs were co-cultured with MSCs for 72 h. Representative images of EdU^+^LEPCs and statistical analysis. ***p < 0.001 *versus* LEPCs group; **(D)** ELISA for IGF-1 in the culture medium of MSCs or LEPCs. ***p < 0.001 *versus* MSCs group; **(E)** Absorbance of LEPCs was detected by CCK-8 after LEPCs were cultured alone, co-cultured with MSCs, or anti-IGF-1 was added to the co-culture system. *p < 0.01 *versus* LEPCs group; #p < 0.01 *versus* MSCs + LEPCs group; Cells were then stained with EdU and statistically analysed for positive cells **(F,G)**. ***p < 0.001 *versus* LEPCs group; ###p < 0.001 *versus* MSCs + LEPCs group; **(H)** CCK-8 experiments to validate the concentration and time dependence of LEPCs on IGF-1. **p < 0.01, ***p < 0.001 *versus* Control group; **(I,J)** Immunoblotting of apoptosis-associated proteins and quantification of blotting intensity after 72 h of IGF-1 (100 ng/mL) treatment of LEPCs, GAPDH was used as a loading control. ***p < 0.001 *versus* LEPCs group; **(K)** Representative images of apoptotic cells detected by flow cytometry; **(L)** Quantification of early, late and total apoptotic percentages in untreated or IGF-1-treated LEPCs. *p < 0.05 *versus* untreated control cells. scale bar 100 µm in B,F; MSCs, mesenchymal stem cells; LEPCs, lymphatic endothelial progenitor cells; IGF-1,insulin-like growth factor 1.

Using transwell chambers to co-culture MSCs and LEPCs for different times, CCK-8 showed that the absorbance value (OD value) of LEPCs co-cultured with MSCs was higher than that of the LEPCs alone group (Figure 3A). The EdU staining results also showed that MSCs under co-culture conditions could promote the proliferation of LEPCs (Figures 3B,C).

ELISA results showed that IGF-1 levels were significantly higher in MSCs medium than in LEPCs medium (Figure 3D). Both CCK-8 results (Figure 3E) and EdU results (Figures 3F,G) showed that blocking IGF-1 in the co-culture system with 10 μg/mL anti-IGF-1 neutralising antibody partially reversed the pro-proliferative effect of MSCs on LEPCs. Subsequently, we treated LEPCs with different concentrations of IGF-1, and the results showed that IGF-1 promoted the proliferation of LEPCs in a concentration-dependent as well as time-dependent manner (Figure 3H), and we chose 100 ng/mL of IGF-1 for the subsequent experiments.

Western blotting results showed increased levels of Bcl-2 and decreased levels of Bax and Caspase3 expression in LEPCs treated with IGF-1 for 72 h (Figures 3I,J). In addition, the results of flow cytometry showed that IGF-1 reduced the early apoptosis rate and total apoptosis rate of LEPCs (Figures 3K,L). The above results suggest that IGF-1 promotes proliferation and inhibits early apoptosis of LEPCs.

### 3.4 IGF-1 promotes activation of PI3K/Akt/mTOR signalling in LEPCs

**FIGURE 4:**
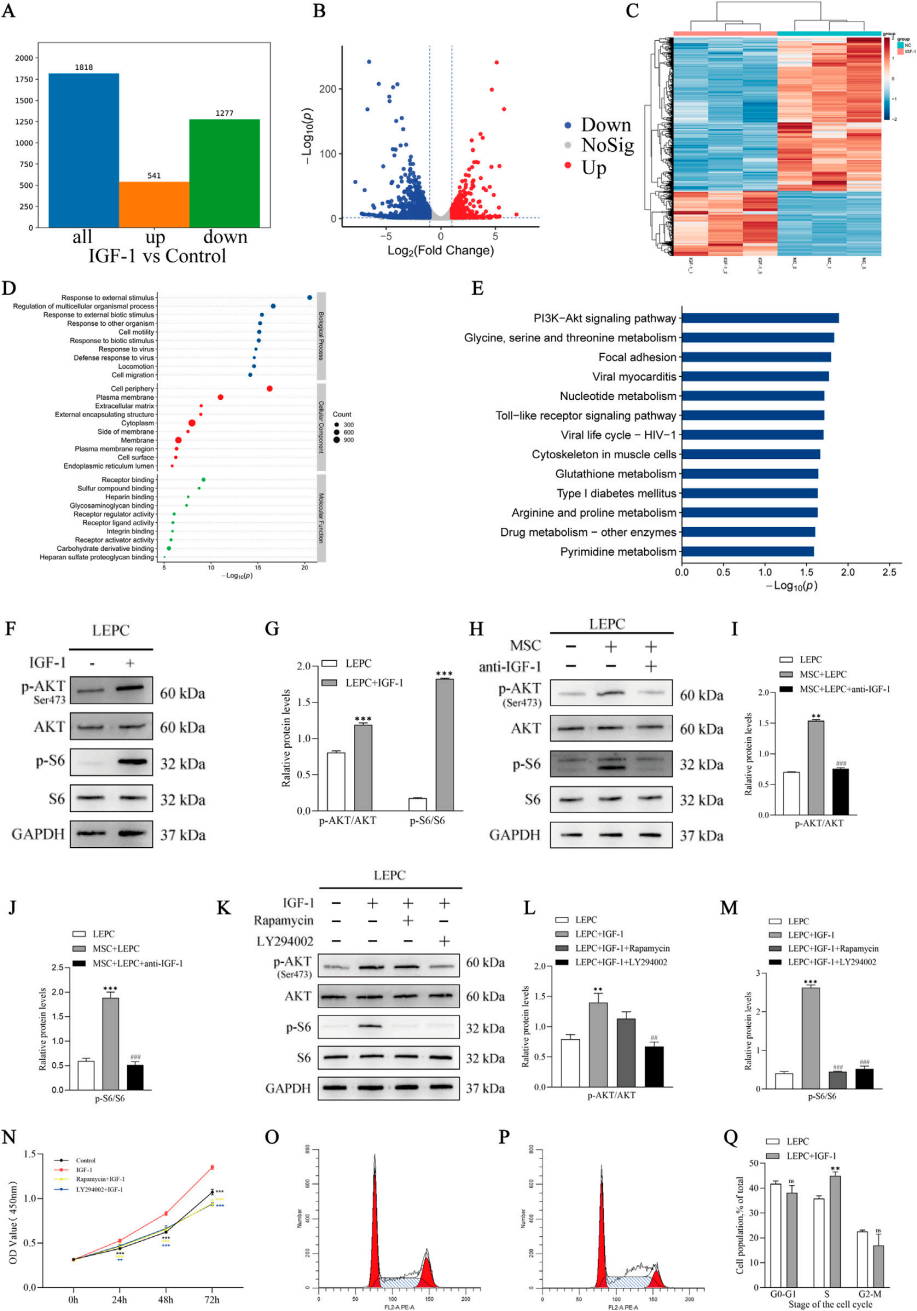
IGF-1 promotes the activation of PI3K/Akt/mTOR signalling in LEPCs. **(A,B)** Differential gene analysis of the IGF-1-treated group compared with the control group, respectively; red dots represent upregulated genes, green dots represent downregulated genes, and grey dots represent non-significant differentially expressed genes. The two vertical dashed lines in the scatterplot are the 2-fold expression difference thresholds; the horizontal dashed line is the p = 0.05 threshold; **(C)** Heatmap of differential gene clustering, the three left columns are samples from the IGF-1 treated group and the three right columns are samples from the control group; red indicates highly expressed genes and blue indicates low expressed genes; **(D)** GO annotation enrichment analysis of DEGs, showing the top 10 terms in terms of enrichment significance under the three branches of biological process (BP), molecular function (MF) and cellular component (CC), Each circle represents a term, and the size of the circle indicates the count; **(E)** KEGG annotation of differential genes in control and IGF-1-treated groups and functional enrichment analysis of the annotation results. The horizontal coordinate is the negative logarithmic transformation of the p-value and the vertical coordinate is the pathway; **(F,G)** Correlation protein blots and blot quantification plots of the PI3K/Akt/mTOR signalling pathway after IGF-1 stimulation of LEPCs, showing representative protein blots from n = 3 independent experiments. ***p < 0.001 *versus* LEPC group; **(H)** Blotting of proteins associated with the PI3K/Akt/mTOR signalling pathway in LEPCs after neutralisation of IGF-1 in the MSCs + LEPCs co-culture system; **(I,J)** Quantification of blot intensity in **(H)** **p < 0.01,***p < 0.001 *versus* LEPC group; ###p < 0.001 *versus* MSC + LEPC group. **(K)** Protein blotting of proteins associated with the PI3K/Akt/mTOR pathway after treatment with a PI3K inhibitor or mTORC1 inhibitor, quantification of blot intensity is shown in **(L,M)**. **p < 0.01,***p < 0.001 *versus* LEPC group; ##p < 0.01, ###p < 0.001 *versus* LEPC + IGF-1 group; **(N)** Proliferation of LEPCs under different stimuli. **p < 0.01,***p < 0.001 *versus* IGF-1 group; Representative flow cytometry cycle results for the LEPC group **(O)** and the LEPC + IGF-1 group **(P)**; The cycle occupancy of the two groups of cells is shown in **(Q)**. ns,p > 0.05; **p < 0.01 *versus* LEPC group.IGF-1,insulin-like growth factor 1; LEPCs, lymphatic endothelial progenitor cells; DEGs, differentially expressed genes; GO,Gene Ontology; KEGG, Kyoyo Encyclopedia of Genes and Genomes.

To explore the potential molecular mechanisms by which IGF-1 promotes the proliferation of LEPCs, transcriptomic studies were performed on LEPCs after 72 h of IGF-1 treatment. The results indicated the presence of 541 upregulated genes and 1277 downregulated genes in the IGF-1-treated group compared to the control group (Figure 4A). The volcano plot shows a symmetrical distribution of differential gene expression between the IGF-1 and control groups (Figure 4B). Meanwhile, the expression levels of the same gene in both sets of samples and the expression patterns of different genes in the same set of samples were clustered (Figure 4C). The genes and gene products of the 2 cell groups were annotated using the GO database and described in terms of Biological Process (BP), Cellular Component (CC) and Molecular Function (MF), the results showed that the most representative subcategory in BP was response to external stimulus, the most representative subcategory in CC was cell periphery, and the most representative subcategory in MF was receptor binding (Figure 4D). KEGG annotation of the differential genes between the comparison groups and functional enrichment analysis showed that a number of pathways, including the PI3K-Akt signalling pathway, were enriched in the IGF-1-treated group (Figure 4E).

Western blotting results showed that the phosphorylation level of Akt, a key protein of the PI3K/Akt pathway, was increased in IGF-1-treated LEPCs, and interestingly, S6, a key protein of the mTOR pathway, was also activated (Figures 4F,G), whereas there was no significant change in the total protein levels of Akt and S6 (Supplementary Figure S3A). Similar changes were also detected in LEPCs co-cultured with MSCs, whereas the phosphorylation-activating effect of MSCs on Akt and S6 was reversed after IGF-1 neutralisation in the co-culture system (Figures 4H–J; Supplementary Figure S3B). We then inhibited the PI3K/Akt/mTOR pathway in LEPCs using the PI3K inhibitor LY294002 (20 µM) (MCE, United States) and the mTOR inhibitor rapamycin (100 nM) (MCE, United States), respectively, and the results showed that the phosphorylation levels of both Akt and S6 were successfully inhibited (Figures 4K–M; Supplementary Figure S3C). In the CCK-8 assay, IGF-1 was found to significantly increase the viability of LEPCs, whereas inhibition of PI3K/Akt/mTOR activity by LY294002 and rapamycin reversed the pro-proliferative effect of IGF-1 (Figure 4N). The above evidence suggests an important role for PI3K/Akt/mTOR signalling in the pro-proliferative effect of IGF-1 on LEPCs.

Another thing that intrigued us was that the KEGG enrichment analysis showed that 12 upregulated genes were enriched to the cell cycle in the IGF-1 treatment group (Supplementary Table S1). By examining the cell cycle, it was found that IGF-1 did affect the cell cycle progression of LEPCs, as evidenced by a decrease in the proportion of cells in the G1 phase and an increase in the proportion of cells in the S phase (Figures 4O,P,Q). This suggests from another angle that the effect of IGF-1 on cell proliferation may also be achieved by affecting the cell cycle.

### 3.5 IGF-1 influences nucleotide metabolism in LEPCs

**FIGURE 5:**
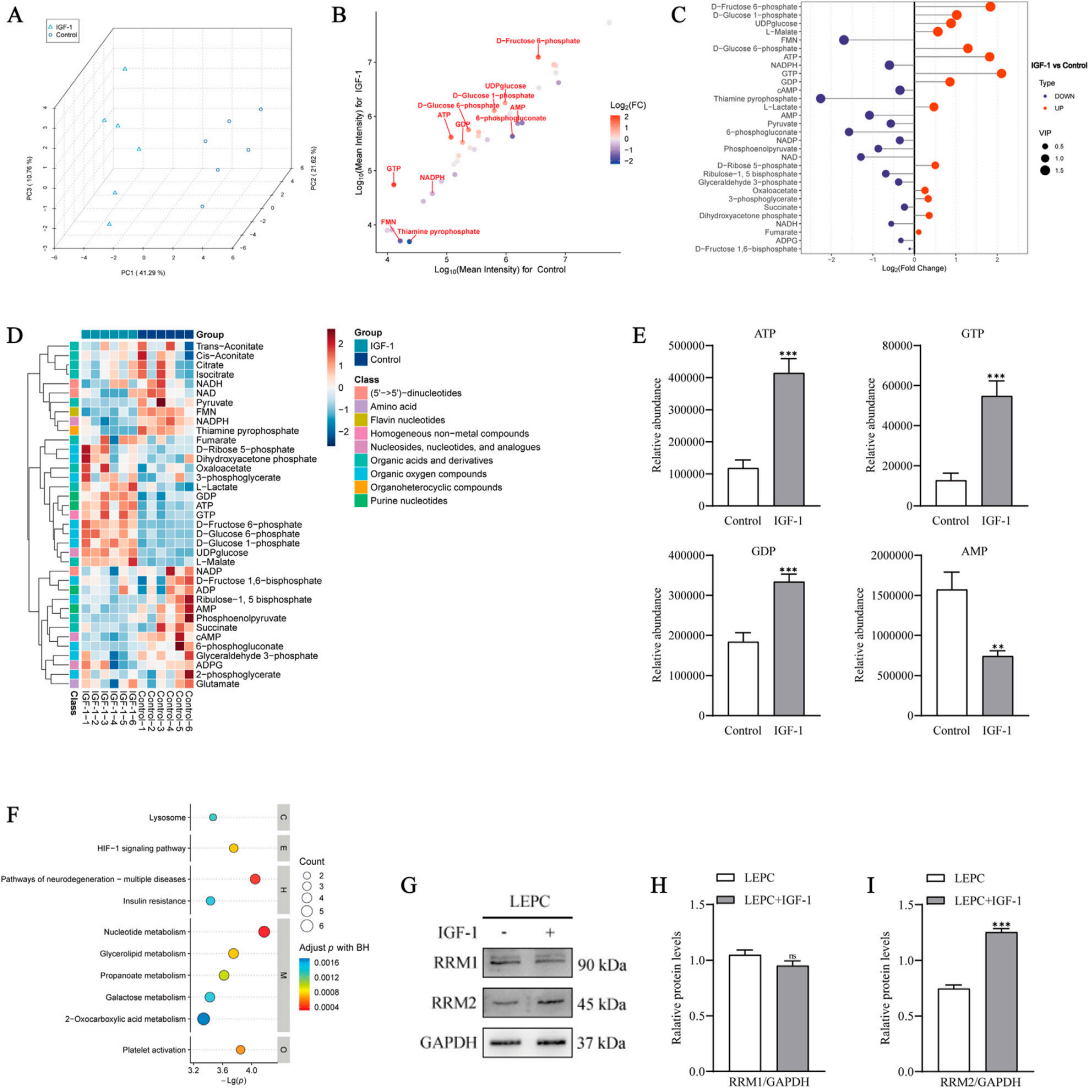
IGF-1 affects nucleotide metabolism in LEPCs. **(A)** Plot of PCA scores for IGF-1 vs control. PCA, principal component analysis. **(B)** Univariate statistical analysis showing the distribution of differentiators between the two groups, significantly different metabolites are labelled, horizontal and vertical coordinates indicate lg log-transformed signal values, and the colour of the dots correlates with FC; **(C)** Metabolite importance analysis showing some differential metabolites with higher importance. The horizontal coordinate is the logarithmic transformation of FC, the vertical coordinate is the metabolites, and the purple and orange dots indicate downregulated and upregulated metabolites, respectively. The size of the dots indicates the VIP value, and variables with higher VIP values are of greater importance; **(D)** Metabolite hierarchical clustering analysis, where redder colours indicate higher relative expression and bluer colours indicate lower relative expression, and Class is metabolite classification information; **(E)** Abundance analysis of the differential metabolites ATP, GTP, GDP and AMP showing the average expression of the metabolites in the two groups, the horizontal coordinates are the two groups and the vertical coordinates are the relative expression. **p < 0.01,***p < 0.001 *versus* control. **(F)** Metabolite KEGG pathway analyses, BH: Benjamini & Hochberg method, with the vertical coordinate letters on the right side representing the different top classifications from top to bottom: Cellular processes; Environmental information processing; Human diseases. Metabolism; Organismal systems; **(G)** Protein blotting associated with nucleotide metabolism reductase after IGF-1 stimulation of LEPCs; **(H,I)** Quantification of imprint intensity in G. ns,p > 0.05; ***p < 0.001 *versus* LEPC group; PCA,principal component analysis; lg,logarithm; FC,fold change; VIP,variable importance for the projection; KEGG, kyoyo encyclopedia of genes and genomes; IGF-1,insulin-like growth factor 1; LEPCs, lymphatic endothelial progenitor cells.

Interestingly, in addition to the PI3K/Akt pathway, KEGG functional enrichment analyses between the IGF-1 group and the control group also enriched for a number of metabolic pathways (Figure 4E), and based on these clues we performed targeted metabolomics analyses of samples from both groups. A significant difference in cellular metabolites after IGF-1 treatment of LEPCs can be seen in the figure (Figure 5A). Univariate analyses of the two groups of differential metabolites were performed to screen out some potential marker metabolites (Figure 5B). Analysis of metabolite importance revealed that some metabolites including GTP, ATP were significantly upregulated in the IGF-1 group (Figure 5C). In order to more comprehensively visualise the relationship between the two groups of samples and the differences in metabolite expression patterns, we examined the stability of metabolite expression in samples within groups by hierarchical clustering of samples from each group using qualitative metabolite expression, which showed a steady increase in the expression of multiple metabolites in the IGF-1 group (Figure 5D). Significantly different metabolite analyses between the two groups showed that seven metabolites were upregulated and five metabolites were downregulated in the IGF-1-treated group compared with the control group (Supplementary Table S2). We analysed the average expression of the differential metabolites ATP, GTP, GDP and AMP between the two groups (Figure 5E), and the expression of other differential metabolites is provided in the Supplementary Material (Supplementary Figure S4), which showed that the expression of the nucleotide metabolites ATP, GTP and GDP was significantly higher in the IGF-1-treated group than in the control group, whereas the expression of AMP was significantly lower than in the control group. Finally we performed KEGG pathway analysis on the differential metabolites of the two groups and found that nucleotide metabolism was enriched in metabolic classification (Figure 5F).

Based on this result, we used protein immunoblotting to detect the catalytic subunits RRM1 and RRM2 of ribonucleotide reductase (RNR), a key enzyme in nucleotide metabolism, in IGF-1-treated *versus* control groups. The results showed that IGF-1 increased the expression of RRM2 in the cells, while there was no significant difference in the changes of RRM1 (Figures 5G–I). In conclusion, these results suggest that IGF-1 somehow affects the level of nucleotide metabolism in LEPCs.

### 3.6 Combined transplantation of MSCs and LEPCs alleviates hindlimb lymphedema in mice

**FIGURE 6:**
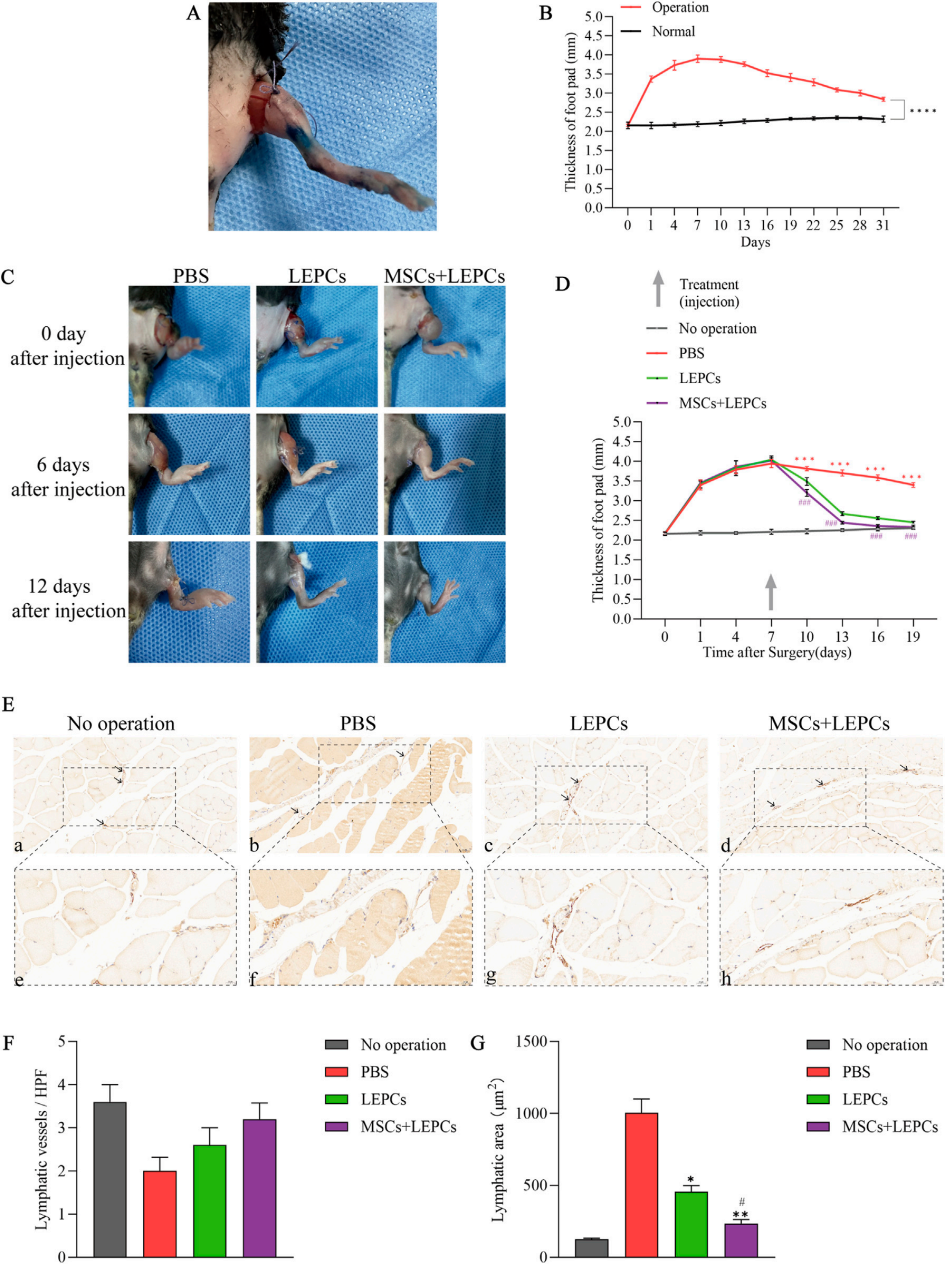
Combined transplantation of MSCs and LEPCs attenuates hindlimb lymphedema in mice. **(A)** Images of mouse hind limbs 1 day after surgery; **(B)** footpad thickness of surgical limbs *versus* normal limbs in mice,****p < 0.0001; **(C)** Images of hindlimb oedema in mice at 0, 6 and 12 days after cell transplantation, each set of images from the same mouse; **(D)** Measurement of footpad thickness in each group of mice after cell transplantation,***p < 0.001 *versus* other groups; ###p < 0.001 *versus* LEPCs group; **(E)** Immunohistochemistry using the lymphatic endothelial marker LYVE-1 in mouse hindlimb samples 12 days after cell transplantation; lymphatic vessels are brown (arrowheads); scale bar 50 µm in a-d,scale bar 20 µm in e-h; **(F,G)** Analysis of the number of lymphatic vessels and the total area of lymphatic vessels in the immunohistochemical results. *p < 0.05, **p < 0.01 *versus* PBS group; #p < 0.05 *versus* LEPCs group. MSCs, mesenchymal stem or stromal cells; LEPCs, lymphatic endothelial progenitor cells; LYVE-1,lymphatic vessel endothelial hyaluronan receptor-1; HPF:high-power field.

The mice had no bleeding or infected conditions 1 day after hindlimb surgery (Figure 6A). We determined the extent of hindlimb oedema by regularly measuring and comparing the footpad thickness of the surgical limb (left hindlimb) with that of the normal limb (right hindlimb) in mice, and showed that the surgery could successfully induce and maintain hindlimb oedema for at least 31 days, with the most significant oedema at postoperative day 7 (Figure 6B).

We next evaluated the therapeutic effects of MSCs and LEPCs on lymphedema and showed that the recovery of hindlimb oedema was slow in negative control mice injected with PBS alone, whereas hindlimb oedema was significantly alleviated in mice transplanted with LEPCs alone or in combination with MSCs and LEPCs (Figure 6C), as confirmed by changes in the thickness of the foot pads (Figure 6D). Notably, combined transplantation of the 2 cell types resulted in greater relief of oedema than transplantation of LEPCs alone, and the oedematous hindlimb largely returned to normal 12 days after combined cell transplantation.

To observe changes in lymphatic vessels within the limbs of each group of mice, we labelled lymphatic vessels in the tissues by immunohistochemistry using the lymphatic vessel endothelial-specific marker LYVE-1. The results showed that the expression of lymphatic vessels was present in all groups, but there was no significant difference in their number (Figures 6E,F). However, analysis of the total area of lymphatic vessels in each group showed that the area of LYVE-1^+^ lymphatic vessels in the PBS group was significantly larger than the area in the unoperated group, suggesting the presence of lymphatic fluid stagnation (Figure 6G). In comparison, the lymphatic vessel area in the cell transplantation groups was significantly smaller than that in the PBS group, and the lymphatic vessel area in the group transplanted with MSCs in combination with LEPCs was smaller than that in the group transplanted with LEPCs alone.

## 4 Discussion

Since their discovery, MSCs have played a role in several disease areas where they are able to homing to damaged tissues and provide various growth factors and regenerative molecules, thus playing a therapeutic and tissue regenerative role (Ullah et al., 2019). The multidirectional differentiation capacity of MSCs allows them to replace damaged cells and also to interact with other cells by secreting a variety of cytokines and growth factors (Fu et al., 2017), making them ideal seed cells for stem cell therapy.

In secondary lymphedema, MSCs can not only differentiate into lymphatic endothelial cells, but also secrete lymphangiogenic factors such as VEGF-C and bFGF to promote lymphatic endothelial cell proliferation, migration and differentiation, and subsequently alleviate lymphedema (Zhan et al., 2015; Yang et al., 2018; Ahmadzadeh et al., 2020; Jørgensen et al., 2021). VEPCs, also known as EPCs, can differentiate into mature vascular endothelial cells and participate in vascular neogenesis. In 2003, Salven P et al. (Salven et al., 2003) extracted LEPCs that differed from EPCs in surface markers, differentiation propensity and biological function, and such cells were considered to be a lymphoid subpopulation of EPCs. In recent years, LEPCs have been suggested by scholars to be involved in developmental and postnatal lymphangiogenesis (Park et al., 2011; Kazenwadel and Harvey, 2018). LEPCs isolated from human cord blood can differentiate into lymphatic endothelial cells and form lymphatic capillaries in Matrigel induced by VEGF-C (Tan et al., 2014).

Given that both MSCs and LEPCs have the ability to differentiate into lymphatic endothelial cells, the interactions and synergistic effects between these two stem cells have piqued our interest. In the present study, we found that MSCs could promote the proliferative capacity of LEPCs by indirect co-culture of the 2 cell types, a phenomenon suggesting that MSCs may provide the microenvironment necessary to support the proliferation of LEPCs by secreting cytokines or growth factors. Among the many factors secreted by MSCs, although VEGF-C is considered a key mediator of lymphangiogenesis, it has been shown that MSC-CM is superior to rVEGF-C alone in promoting lymphangiogenesis (Takeda et al., 2015), suggesting that other growth factors secreted by MSCs may also have a pro-lymphangiogenic effect.

Our previous study showed that MSCs secreted IGF-1 to promote the proliferation of EPCs (Hou et al., 2017). IGF-1 generally acts in an autocrine or paracrine manner and can affect the functional activity of many types of cells or tissues (Fu et al., 2007; Lin et al., 2017; Song et al., 2017; Long et al., 2019), is involved in protecting the activity of EPCs and stimulating their cloning (Humpert et al., 2008; Li et al., 2024), and induces lymphangiogenesis *in vivo* (Björndahl et al., 2005). LEPCs as a subpopulation of EPCs, we speculated whether IGF-1 was also involved in promoting LEPC proliferation by MSCs. Elisa results in this study showed that the concentration of IGF-1 in the medium of MSCs was significantly higher than that in the medium of LEPCs. Subsequently, the pro-proliferative effect of MSCs on LEPCs was significantly attenuated after blocking IGF-1 in the co-culture system with a neutralising antibody, suggesting that IGF-1 secreted by MSCs mediates the pro-proliferative effect of MSCs on LEPCs. IGF-1 can act as an anti-apoptotic factor to prevent apoptosis (Li et al., 2020; Lin et al., 2020), and in order to investigate its role in the survival of LEPCs, we examined the expression of apoptosis-related proteins in cells. The results suggest that IGF-1 affects the expression of apoptotic proteins in LEPCs and also reduces their apoptosis rate, but mainly in early apoptosis. As primary cells, LEPCs decline in growth capacity and activity with increasing cell generations, and the inhibitory effect of IGF-1 on apoptosis may delay this process, thereby increasing cell survival.

It is known that the PI3K/Akt pathway is the main signalling pathway of the insulin signalling pathway and that IGF-1 can regulate cell proliferation by activating this pathway (Lin et al., 2017; Han et al., 2021). In the present study, after stimulation of LEPCs with IGF-1, the phosphorylation levels of Akt and S6 were significantly increased, indicating that the PI3K/Akt/mTOR pathway was activated, whereas the use of pathway inhibitors significantly attenuated the pro-proliferative effect of IGF-1 on LEPCs, reflecting the involvement of the PI3K/Akt/mTOR pathway in IGF-1-induced proliferation of LEPCs. In addition, flow cytometry results showed that IGF-1 affected the cell cycle in LEPCs, and considering that the Akt pathway can regulate cell cycle progression while affecting cellular DNA replication (Shaw and Cantley., 2006), the cell cycle changes in LEPCs may also be related to the activation of the IGF-1 downstream pathway.

On the other hand, IGF-1 also affected the metabolic levels of LEPCs. Significant changes in the nucleotide levels of LEPCs after IGF-1 stimulation were considered to be a result of their increased metabolic activity and promoted nucleotide synthesis. Meanwhile, IGF-1 affects changes in the levels of the nucleotide reductase RRM2 in cells, and transcriptional activation of RRM2 can significantly stimulate the activity of RNR to ensure an adequate supply of dNTPs for DNA replication (Nordlund and Reichard., 2006; Aye et al., 2015), we reasoned that the IGF-1-induced increase in RRM2 levels was due to the active proliferative state of the cells, and that LEPCs would adjust their nucleotide metabolism to meet proliferative needs when stimulated by IGF-1. Overall, these changes suggest that IGF-1 may regulate the function of LEPCs by promoting energy metabolism and influencing nucleotide metabolism pathways, and the deeper mechanisms involved need to be further investigated.

Relevant studies have shown that both MSCs and LEPCs have some lymphangiogenic potential, and lymphedema in animal models can be alleviated by cell transplantation (Conrad et al., 2009; Buttler et al., 2014; Campbell et al., 2021). In this study, we injected MSCs and LEPCs at the site of lymphedema in mice to evaluate the therapeutic effect of combined transplantation of the two stem cells for lymphedema. Combined transplantation of the 2 cell types was more effective than transplantation of LEPCs alone in reducing swelling in the hind limbs of mice. The key to the treatment of lymphedema lies in the repair and reconstruction of lymphatic vessels and the restoration of function, Frueh et al. used LYVE-1 to assess lymphatic vessel area to understand the extent of lymphatic stasis (Frueh et al., 2022). In the present study, the results of immunohistochemistry showed a reduction in the area of lymphatic vessels in the limbs of the mice in the combined transplantation group, which to some extent reflects the fact that cell transplantation can promote the recovery of lymphatic vessel dilation due to the blockage of lymphatic fluid. However, there was no significant difference in the number of lymphatic vessels between the groups. We speculate that after transplantation into the mice, the cells were unable to rebuild the lymphatic vessels within a short period of time due to other factors such as immune response and inflammatory factors in the oedema sites, but they may be able to alleviate the lymphedema by releasing pro-lymphopoietic factors or by differentiating into lymphatic endothelial cells to improve the function of the lymphatic vessels and promote lymphatic circulation. Although the therapeutic efficacy of MSCs in combination with LEPCs in lymphedema was initially demonstrated, the *in vivo* experiments still have some limitations, and it needs to be further verified whether MSCs exert the same pro-proliferative effect on LEPCs *in vivo* and whether IGF-1 mediates this process. In addition, the efficiency of lymphatic drainage can be examined to clarify the recovery of lymphatic vessel function, and perhaps in the future, fluorescent tracers or other experimental methods can be used to monitor the flow of lymphatic fluid to assess lymphatic vessel function. The method of observing the area and number of lymphatic vessels in this study is convenient and quick, but it can be supplemented with other experimental methods to better observe the morphologic changes of lymphatic vessels, thus providing further insights into the efficacy of MSCs and LEPCs in the treatment of lymphedema.

## 5 Conclusion

In conclusion, our study initially explored the role of IGF-1 secretion from MSCs in regulating the biological behaviour of LEPCs. The pro-proliferative effect of IGF-1 on LEPCs is mediated by the PI3K/Akt/mTOR pathway, and IGF-1 inhibits apoptosis, promotes cell cycle progression and affects nucleotide metabolism in LEPCs. Combined transplantation of MSCs and LEPCs is superior to transplantation of LEPCs alone in mouse lymphedema limbs. An in-depth understanding of the interactions between MSCs and LEPCs will not only help to develop better stem cell therapeutic strategies, but also optimise current cell-based approaches to lymphedema treatment.

## Data Availability

The datasets presented in this study can be found in online repositories. The names of the repository/repositories and accession number(s) can be found in the article/Supplementary Material.
