# Supplementary material for: IGF-1 secreted by mesenchymal stem cells affects the function of lymphatic endothelial progenitor cells: a potential strategy for the treatment of lymphedema
Source: Front Genet. 2025 May 21;16:1584095. doi: 10.3389/fgene.2025.1584095 (PMC12133893; doi:10.3389/fgene.2025.1584095)
Supplement: Supplementary file 1 [file Supplementaryfile1.docx]

Supplementary Material

# Supplementary Figures and Tables

## Supplementary Figures


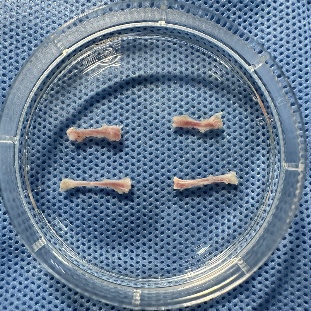


**Figure S1:** Femur and tibia of the hind limb of a mouse.


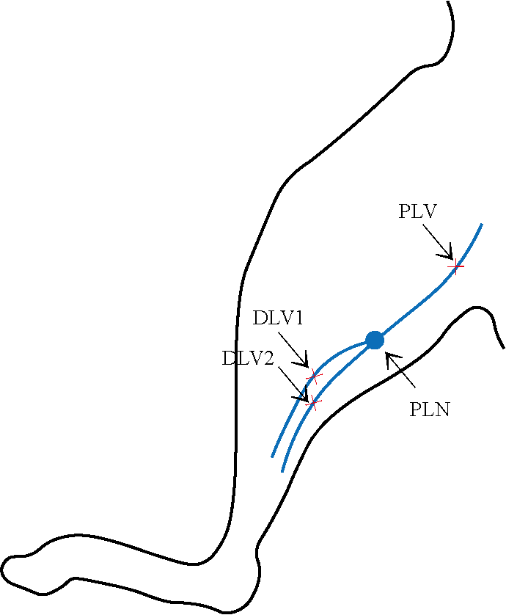


**Figure S2:** Schematic diagram of lymph nodes and lymphatic vessels in the left hind limb of mice (Dorsal view).

PLN: the popliteal lymph node; PLV: the one lymph vessel proximal to the lymph node; DLV1 and DLV2: the two lymph vessels distal to the lymph node. × represents the site of ligation.


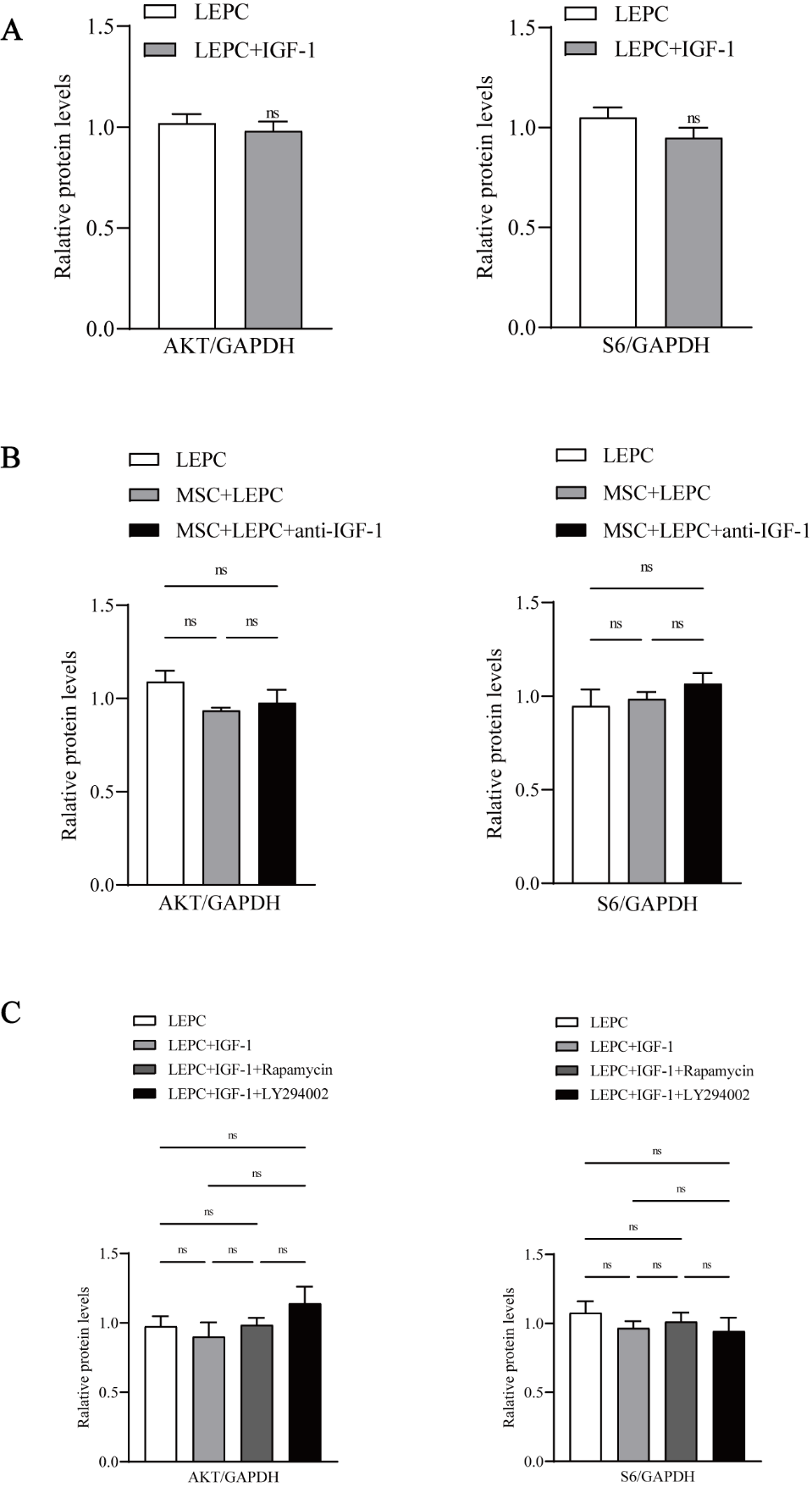


**Figure S3.** Results of blotting quantification of Akt and S6, proteins related to the PI3K/Akt/mTOR signalling pathway, in LEPCs. (**A**) Immunoblot quantification of total proteins of Akt and S6 in LEPCs. ns p > 0.05 versus LEPC group; (**B**) Immunoblot quantification of total proteins of Akt and S6 in LEPCs after blocking of IGF-1 in the co-culture system of MSCs+LEPCs. ns p > 0.05; (**C**) After treatment with a PI3K inhibitor or mTORC1, quantification of total protein blotting intensity of Akt and S6 in LEPCs after treatment with PI3K inhibitor or mTORC1 inhibitor, ns p > 0.05. LEPCs, lymphatic endothelial progenitor cells ;IGF-1, insulin-like growth factor 1.


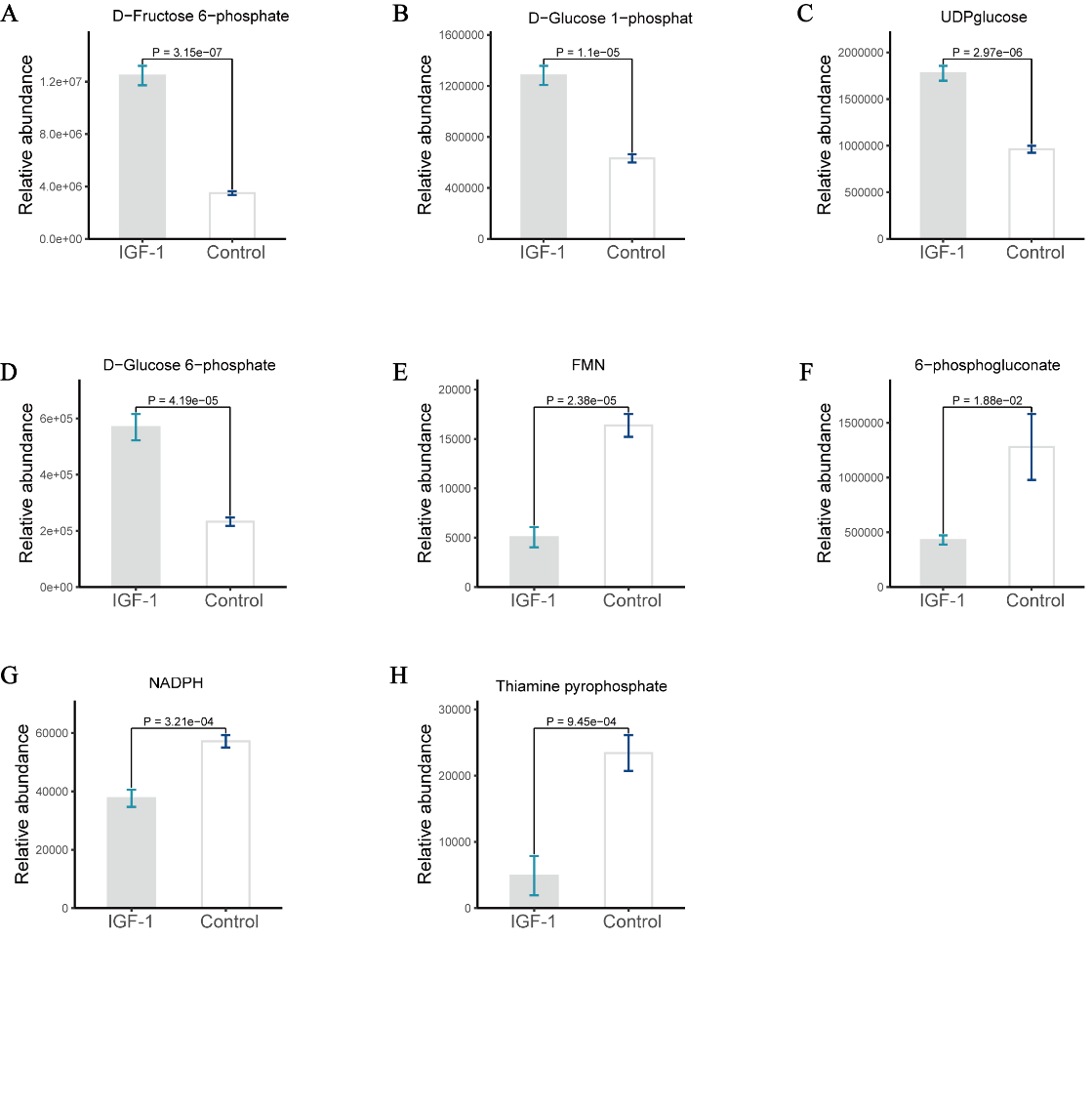


**Figure S4.** Abundance analysis of differential metabolites between IGF-1 treatment and control groups, showing the average expression of different differential metabolites in the two groups, with the horizontal coordinates being the two groups and the vertical coordinates being the relative expression.LEPCs, lymphatic endothelial progenitor cells;IGF-1, insulin-like growth factor 1.

## Supplementary Tables

**Table S1.** Insulin-like growth factor 1（IGF-1）-up-regulated genes enriched in the cell cycle.

| KEGG | Genes |
| --- | --- |
| cell cycle | TRIP13；CDC45；CCND1；CDC20；PKMYT1；GADD45G；CCNA1；ESPL1；MYC；CDCA5；CDC25A；PLK1 |

**Table S2.** Significantly Different Metabolites (IGF-1 vs Control).

| Metabolite.name | Fold Change(FC) | *P*.value | Significant |
| --- | --- | --- | --- |
| ATP | 3.519509145 | 0.000185479 | UP |
| GTP | 4.292313058 | 0.000450295 | UP |
| GDP | 1.813511559 | 0.000488855 | UP |
| D-Fructose 6-phosphate | 3.565553033 | 3.15241E-07 | UP |
| D-Glucose 1-phosphate | 2.030523281 | 1.10367E-05 | UP |
| UDPglucose | 1.84744597 | 2.97205E-06 | UP |
| D-Glucose 6-phosphate | 2.445817708 | 4.1883E-05 | UP |
| AMP | 0.47211141 | 0.004515317 | DOWN |
| FMN | 0.308241221 | 2.38294E-05 | DOWN |
| NADPH | 0.659015146 | 0.000320792 | DOWN |
| Thiamine pyrophosphate | 0.208918101 | 0.000944676 | DOWN |
| 6-phosphogluconate | 0.335623999 | 0.018773602 | DOWN |
